# Use of aminoglycoside 3′ adenyltransferase as a selection marker for *Chlamydia trachomatis* intron-mutagenesis and in vivo intron stability

**DOI:** 10.1186/s13104-015-1542-9

**Published:** 2015-10-15

**Authors:** Nicole M. Lowden, Laxmi Yeruva, Cayla M. Johnson, Anne K. Bowlin, Derek J. Fisher

**Affiliations:** Department of Microbiology, Southern Illinois University, 1125 Lincoln Drive, Carbondale, IL 62901 USA; Departments of Pediatrics, Arkansas Children’s Hospital Research Institute, Arkansas Children’s Nutrition Center, University of Arkansas for Medical Sciences, Little Rock, AR 72202 USA

**Keywords:** *Chlamydia trachomatis*, Group II intron, Mutagenesis, *incA*, *rsbV1*, *aadA*

## Abstract

**Background:**

*Chlamydia* spp. are obligate, intracellular bacteria that infect humans and animals. Research on these important pathogens has been hindered due to a paucity of genetic tools. We recently adapted a group II intron (GII) mutagenesis platform for creation of ampicillin-selectable gene insertions in *C. trachomatis* L2. The aims of this study were: (1) to assess the stability of the intron-insertion in an in vivo infection model to gauge the efficacy of this genetic tool for long term animal studies and (2) to expand upon the utility of the method by validating a second selection marker (*aadA*, conferring spectinomycin resistance) for mutant construction.

**Results:**

Intron stability was assessed using a mouse vaginal tract infection model with a *C. trachomatis* L2 434/Bu *incA*::GII(*bla*) mutant. Infections were performed in the absence of selection and isolates shed into the vaginal tract were isolated and expanded in cell culture (also without selection). PCR and inclusion phenotype analysis indicated that the intron was stable for at least 27 days post-infection (at which point bacteria were no longer recovered from the mouse). The aminoglycoside 3′ adenyltransferase (*aadA*) gene was used to create a spectinomycin-selectable GII intron, facilitating the construction of an *incA*::GII[*aadA*] *C. trachomatis* L2 insertion mutant. Both the GII(*aadA*) intron and our previously reported GII(*bla*) intron were then used to create an *incA*::GII(*aadA*), *rsbV1*::GII(*bla*) double mutant. Mutants were confirmed via PCR, sequencing, inclusion morphology (*incA* only), and western blot.

**Conclusions:**

The stability of the intron-insertion during in vivo growth indicates that the GII-insertion mutants can be used to study pathogenesis using the well-established mouse infection model. In addition, the validation of an additional marker for mutagenesis in *Chlamydia* allows for gene complementation approaches and construction of targeted, double mutants in *Chlamydia*. The *aadA* marker also could be useful for other genetic methods. Collectively, our results expand upon the rapidly growing chlamydial genetic toolkit and will aid in the implementation of studies dissecting the contribution of individual genes to infection.

**Electronic supplementary material:**

The online version of this article (doi:10.1186/s13104-015-1542-9) contains supplementary material, which is available to authorized users.

## Background

*Chlamydia* spp. infect a wide variety of animals and cause serious disease in humans including pneumonia, trachoma (an ocular infection), and sexually transmitted infections (STI) [1]. The latter are caused by *C. trachomatis* and represent the most prevalent reportable bacterial STI in both the United States and world-wide [2, 3]. These obligate, intracellular bacterial pathogens have a unique developmental cycle transitioning between the extracellular, infectious form known as the elementary body (EB) and the intracellular, replicative form referred to as the reticulate body (RB) [4]. Differentiation initiates after cell surface binding and internalization of the EB into a host-derived membrane vacuole termed an inclusion. Within the inclusion the EB differentiates into the RB, which divide by binary fission. After ~20 h post-infection, RBs asynchronously differentiate back into EBs and after 40–72 h post-infection the EBs escape from the cell via cell lysis or inclusion extrusion [5]. Despite their significant negative impact on agriculture and human health and their intriguing physiology, research on *Chlamydia* had been hindered due to the limited number of available genetic tools [6, 7].

In 2011, Wang et al. described a robust chemical transformation protocol and antibiotic-selection conditions that enabled the generation of recombinant *Chlamydia trachomatis* [8]. Since this seminal publication, a variety of recombinant plasmids platforms (based on the native chlamydial cryptic plasmid) have been developed to enable expression of foreign and recombinant genes in *Chlamydia* spp. [9–16]. In addition, chemical-based mutagenesis approaches have been leveraged for use in forward and reverse genetic approaches [17–19]. To complement these approaches, we recently modified the TargeTron mutagenesis system to allow for creation of chromosomal gene insertion mutants in *Chlamydia trachomatis* [20]. This methodology employs a mobile GII intron that can be “targeted” to genes of interest by altering DNA sequences within the 5′ region of the intron via PCR-based mutagenesis [21]. This mutagenesis approach has been utilized for construction of insertion mutants in a variety of Gram-negative and Gram-positive bacteria [22].

Our first generation *bla*-marked intron platform had a number of limitations. Firstly, the *bla*-resistance marker may not be used in the *C. trachomatis* genital serovars D-K as β-lactams (as an alternative therapy) are still a suggested form of treatment for pregnant women (*bla* is subsequently prohibited for use in these strains by the National Institutes of Health Guidelines for Research Involving Recombinant DNA Molecules). Secondly, use of the *bla*-marker limits gene complementation approaches as the majority of chlamydial shuttle vectors utilize the *bla*-marker for plasmid selection and maintenance. Thirdly, use of a single marker limits the number of insertions that may be made in a single bacterium. Finally, while the intron appears stable over multiple passages in cell culture it was unknown whether the intron would be stable in the absence of selection in an extended-period (~1 month) animal model. To address the known limitations and potential problem of in vivo stability, we sought to identify alternative selection markers and to assess the stability of the intron-insertion in a mouse infection model. Validation of new markers for use in *Chlamydia* would expand the growing chlamydial molecular tool kit and allow for experiments querying the role of specific genes in pathogenesis under conditions fulfilling Molecular Koch’s postulates [23]. In addition, demonstration of intron-insertion stability in vivo has implications not just for chlamydial research, but also for other pathogens in which TargeTron-generated mutants are tested in animal models of disease.

## Results and discussion

*In vivo stability of the intron insertion* While cell culture growth experiments with *Chlamydia* allow for the interrogation of numerous hypotheses, animal infection models remain critical for studying innate and adaptive immune responses to infection. Previous animal infection studies with other bacteria carrying GII intron-insertions have been performed [24–29]. However, to the best of our knowledge, intron stability was either not assessed in those studies or the study was short term (less than 1 week). Prior to developing alternative markers for mutant selection in *C. trachomatis*, we wanted to ensure that the intron-insertion would be stable in an animal infection model (the primary concern was loss of the intron from the marked-gene resulting in reconstitution of a wild type genotype).

**Fig. 1:**
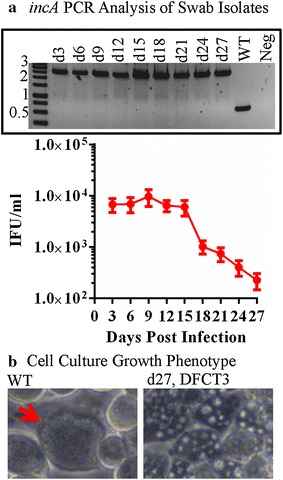
Assessment of intron stability in vivo using a mouse infection model. BALB/c mice were infected intravaginally with DFCT3 (*incA*::GII[*bla*]) in the absence of ampicillin and swabs were taken at 3 day intervals to measure EB titers (IFU/ml), panel **a** [19]. To measure intron-stability, swabs were used to infect mouse L2 cells in the absence of ampicillin and serially passaged to obtain DNA for *incA* PCR (reaction 1, Table 1) and to assess inclusion morphology. Representative *incA* PCR results are shown in panel **a**. In panel **b**, representative phase contrast micrographs (×400) are shown for cells infected at an MOI of ~5 with the wild type strain or with an expansion sample from a day 27 post-infection swab. The *red arrow* highlights a wild type inclusion

**Fig. 2:**
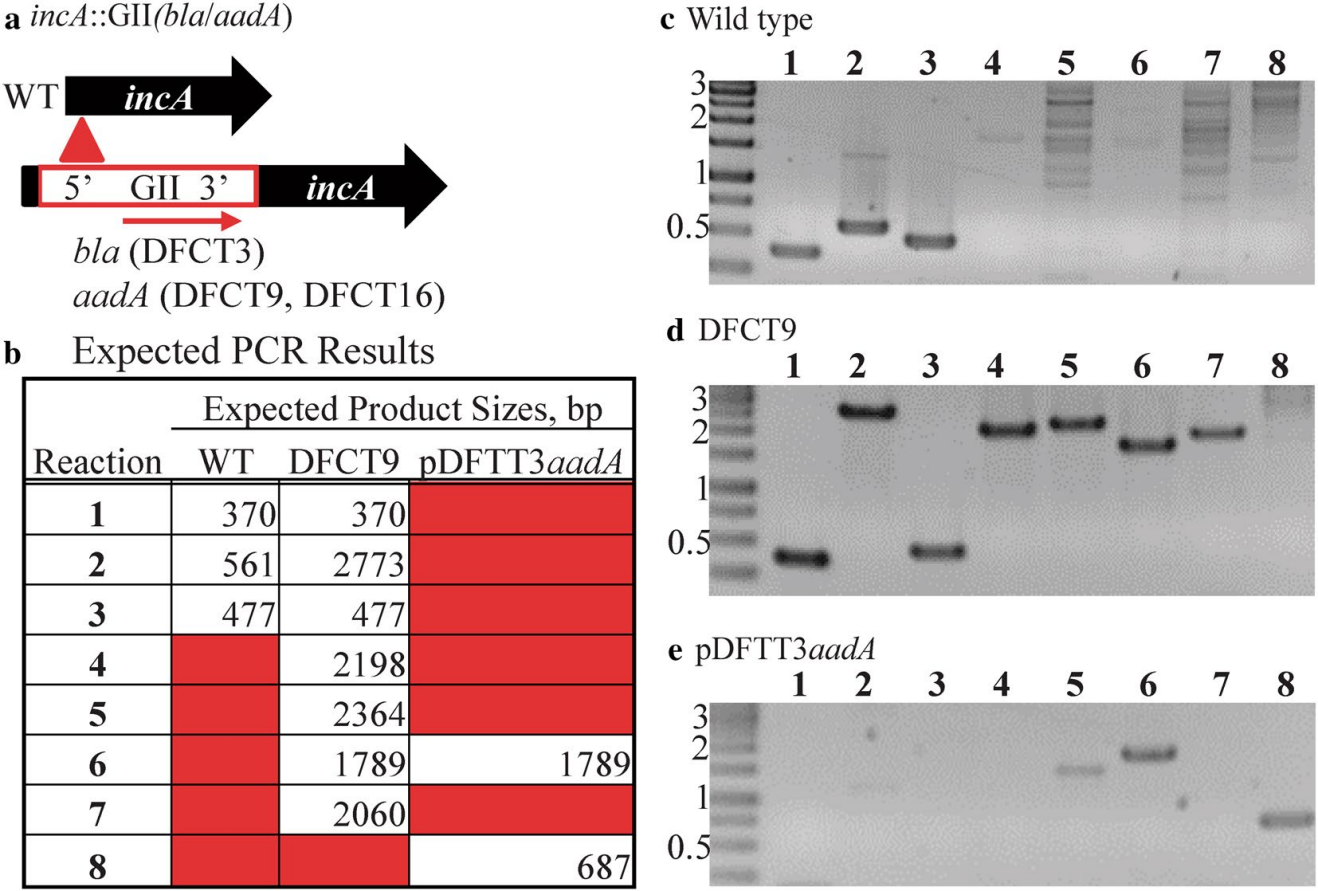
PCR validation of DFCT9, an *incA* insertion mutant created using the GII(*aadA*) spectinomycin-selection cassette. EBs were transformed with pDFTT3*aadA* and serially passaged with spectinomycin selection. Mutants were plaque purified and a single plaque was expanded for PCR and phenotype analysis (shown in Figs. 5, 6). The insertion locus map is shown in panel **a** (DFCT3 is from [19] ) with the intron highlighted in red. The intron is inserted in a sense orientation to *incA*. The GII(*aadA*) intron in vector pDFTT3*aadA* was used to create the *incA* mutation in both DFCT9 and DFCT16 (Fig. 4). Expected PCR product sizes are listed in panel **b**. The primers used and reaction descriptions are listed in Table 1. PCR products were run on 0.8 % agarose gels, stained with ethidium bromide, and visualized with a UV light source. Molecular weight markers (kbp) are shown to the left of each gel. Images were inverted to improve contrast. PCR results are shown for the wild type strain, DFCT9, and pDFTT3*aadA* in panels **c**, **d**, and **e**, respectively

To measure intron stability, mice were infected vaginally with the IncA-null strain DFCT3 (*incA*::GII[*bla*]) [20]. IncA is an inclusion membrane protein that mediates the fusion of multiple inclusions into a single inclusion (each infecting EB initially resides in its own inclusion) [30, 31]. Consequently, cells infected at an MOI >1 with IncA-deficient strains exhibit a non-fusogenic inclusion phenotype [20, 31]. DFCT3-infected mice were swabbed at 3 day intervals post-infection. Swabs were titered using the IFU assay and expanded in cell culture for genotype and phenotype analysis (Fig. 1). No beta-lactams were used during infection or cell culture expansion of swab samples. Detectable levels of EBs were shed until day 27 post-infection (Fig. 1a). Intron-presence was assessed using PCR with *incA*-specific primers flanking the intron insertion site. All isolates were positive for the intron (representative results are shown in the Fig. 1a *inset*) based upon the presence of only the larger, insertion-positive PCR product (*incA*-locus map, Fig. 2a). In addition, cells infected with the isolates at an MOI of ~5 formed non-fusogenic inclusions as previously documented for natural and intron-constructed IncA-null strains (Fig. 1b) [20, 31]. These data indicate that the intron is stable throughout the course of infection in the absence of selection, consistent with results obtained from repeated passage of intron-insertion mutants in cell culture [as seen with DFCT3, 4, 9, 13, and 16 (this study and [20])]. A caveat to the PCR-based approach used for assessing *incA*::GII(*bla*) stability would be the inability to detect large genome arrangements in the mutant strain, although this was not a focus of the study. The ability to carry out antibiotic free in vivo experiments is significant as antibiotic usage alters the normal microflora, which can lead to invalid results.

*Validation of the aadA gene as a selection marker in C. trachomatis* After demonstrating stability of the intron-insertion in vivo, we searched for additional drug resistance markers to: (1) allow for the use of TargeTron outside of the LGV serovar (the NIH prohibits the use of *bla* in *C. trachomatis* serovars D-K), (2) permit complementation of mutants using the *bla*-based shuttle vectors (*bsd* and *cat*-based vectors are available, but these vectors are not as numerous or well-studied as the *bla*-based vectors), and (3) allow for construction of mutants with multiple gene disruptions. To test alternative markers, the *bla* cassette was removed from the intron in pDFTT3 and replaced with either *cat* (chloramphenicol resistance), *arr*-*2* (rifampin resistance), or *aadA* (spectinomycin resistance). All introns remained targeted to *incA*.

Mutagenesis was attempted at least four times using the appropriate antibiotic/intron combination. Experiments with the GII(*cat*) construct resulted in no mutants and experiments with the GII(*arr*-*2*) construct only yielded spontaneous rifampin-resistant mutants (data not shown). Rif^r^ strains were not sequenced for mutations in *rpoB* (as previously reported for spontaneous mutants [32]), but were confirmed to be intron negative by PCR (data not shown). In contrast, experiments with the *aadA* marker consistently resulted in Spec^r^, IncA-null strains (the non-fusogenic phenotype was frequently observed at passage P_3_). Mutants were plaque isolated and a single clone, DFCT9 (*incA*::GII[*aadA*]), was chosen for expansion and characterization. Intron insertion into *incA* was validated and mapped using PCR (results are shown in Fig. 2, reactions are described in Table 1) and the *incA*::GII(*aadA*) and wild type loci also were cloned for DNA sequencing analysis. The intron inserted in a sense orientation between base pairs 108 and 109 of the ORF as predicted by the TargeTron algorithm and as previously found with the identically targeted GII(*bla*) intron (p) [20].

**Table 1:** PCR reactions used to map intron insertions in DFCT3, DFCT9, DFCT13, and DFCT16

Reaction	Primers	Target	Notes
**1**	V1F–V1R	*rsbV1*	Confirms intron insertion (primers flank insertion site)
**2**	incAF–incAR	*incA*	Confirms intron insertion (primers flank insertion site)
**3**	hyp08F–hyp08R	cryptic plasmid	Confirms that transformation has not resulted in loss of the native, cryptic plasmid
**4**	incAF–GIIR	Connects the 5′ region of *incA* to the intron	Confirms intron insertion and orientation
**5**	incAR–GIIF	Connects the 3′ region of *incA* to the intron	Confirms intron insertion and orientation
**6**	GIIF–GIIR	Intron	Confirms intron presence
**7**	incAF–aadAR	Connects *incA* to the *aadA* marker	Confirms marker type
**8**	catF–catR	Intron donor plasmid	Confirms loss of intron donor plasmid
**9**	V1F–GIIF	Connects the 5′ region of *rsbV1* to the intron	Confirms intron insertion and orientation
**10**	V1R2–GIIR	Connects the 3′ region of *rsbV1* to the intron	Confirms intron insertion and orientation
**11**	blaR2–VIR	Connects *rsbV1* to the *bla* marker	Confirms marker type
**12**	blaF1–blaR1	Marker	Confirms presence of *bla* marker
**13**	aadAF–aadAR	Marker	Confirms presence of *aadA* marker

**Fig. 3:**
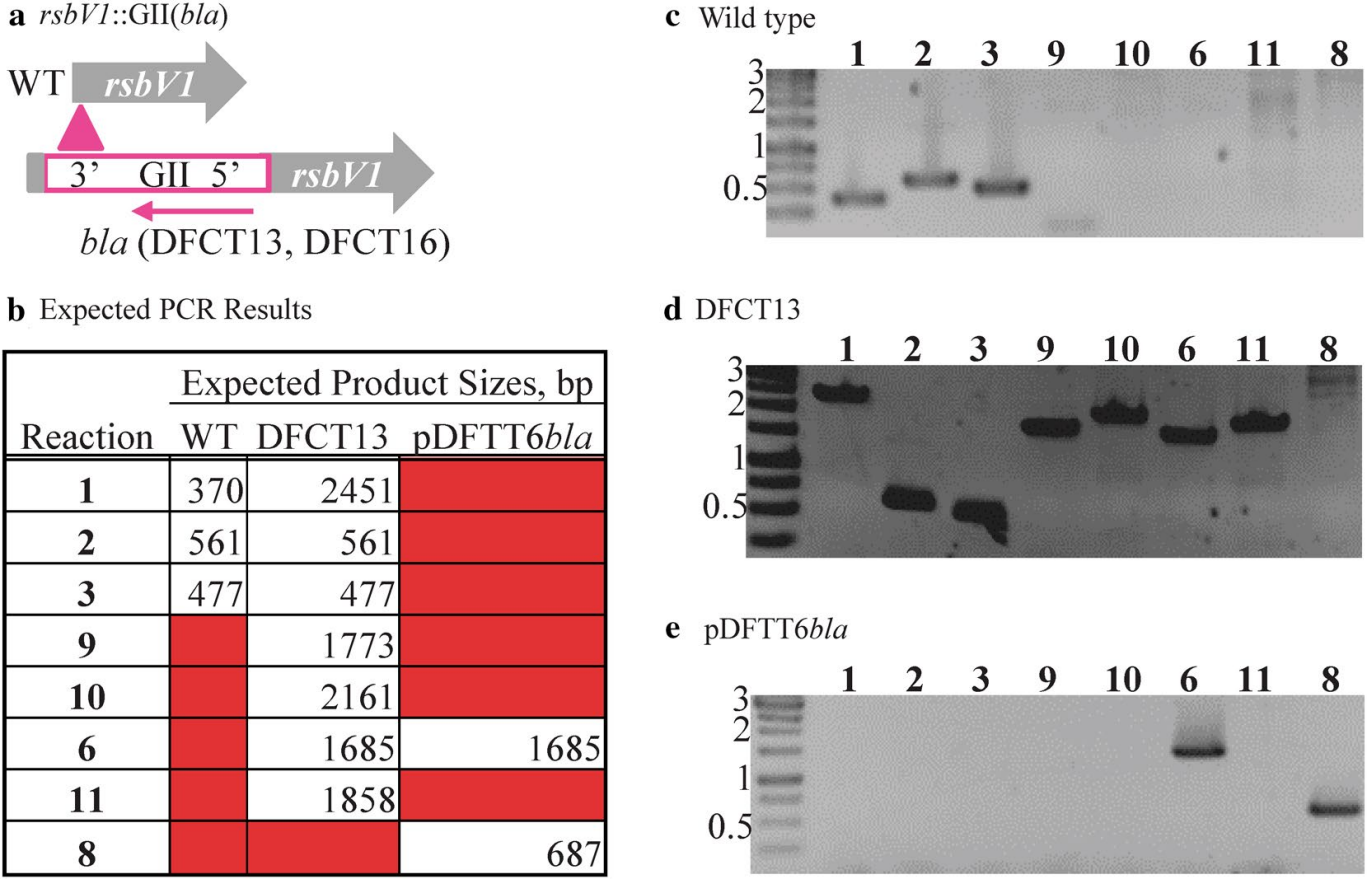
PCR validation of DFCT13, an *rsbv1* insertion mutant created using the GII(*bla*) ampicillin-selection cassette. EBs were transformed with pDFTT6*bla* and serially passaged with ampicillin selection. Mutants were plaque purified and a single plaque was expanded for PCR and phenotype analysis (Figs. 5, 6). The insertion locus map is shown in panel **a** with the intron highlighted in *pink*. The intron is inserted in an anti-sense orientation to *rsbV1*. DNA was isolated from EBs and used for PCR. Expected product sizes are listed in panel **b** and the primers used along with reaction notes are listed in Table 1. PCR products were separated and visualized as described for Fig. 2. PCR results are shown for the wild type strain, DFCT13, and pDFTT6*bla* in panels **c**, **d**, and **e**, respectively

**Fig. 4:**
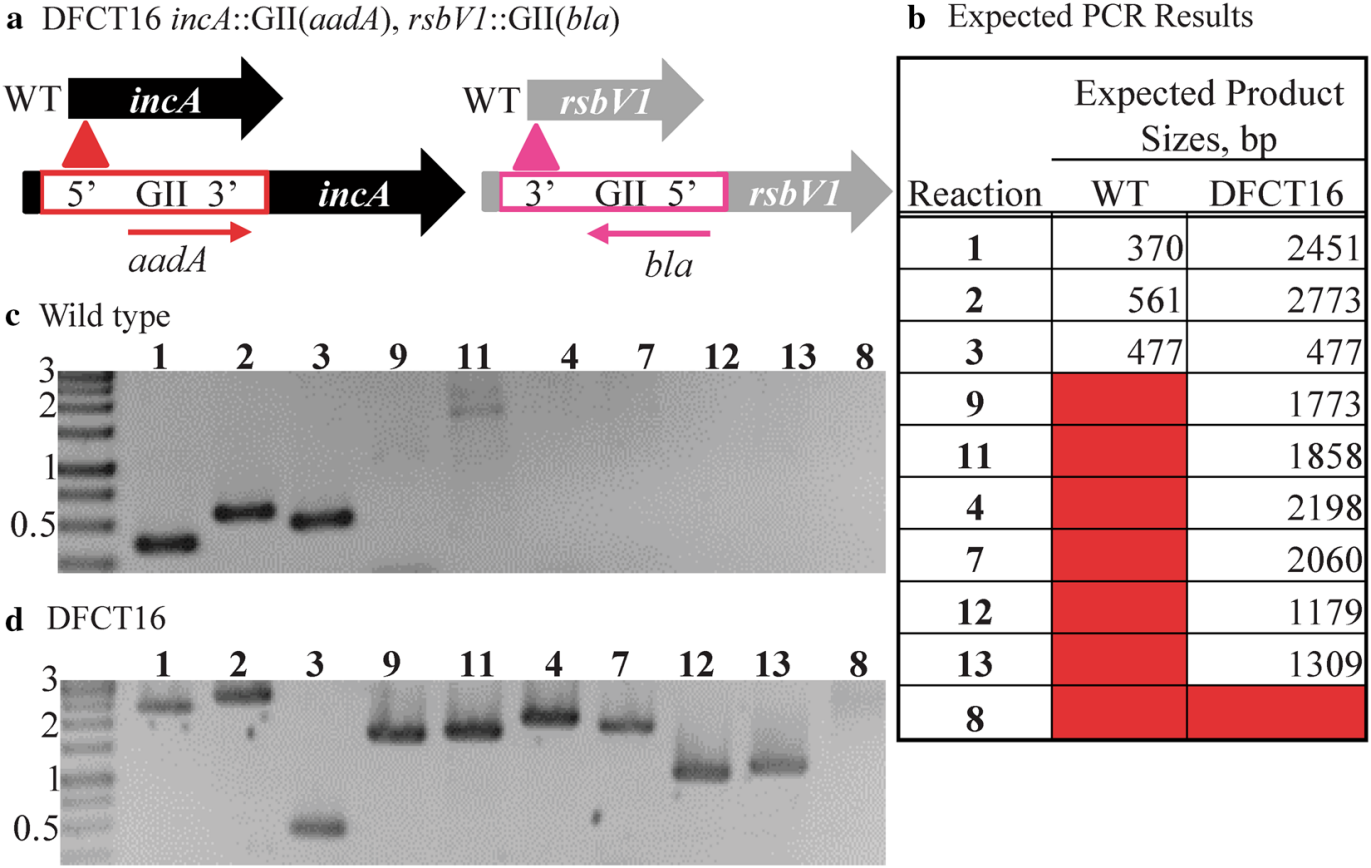
PCR validation of DFCT16, an *rsbv1* and *incA* dual-insertion mutant created using the GII(*bla*) and GII(*aadA*) cassettes. DFCT13 EBs were transformed with pDFTT3*aadA* and serially passaged with spectinomycin selection. Mutants were plaque purified and a single plaque was expanded for PCR and phenotype analysis (Figs. 5, 6). The *incA* and *rsbV1* insertion locus maps are shown in panel **a**. Expected PCR product sizes are listed in panel **b** and the primers used along with reaction notes are listed in Table 1. PCR products were separated and visualized as described for Fig. 2. PCR results are shown for the wild type strain and DFCT16 in panels **c** and **d**, respectively

After validating the *aadA* marker for mutagenesis, we sought to create a double-insertion mutant using the GII(*bla*) intron targeted to *rsbV1* (pDFTT6*bl*a) and the GII(*aadA*) intron targeted to *incA*. The *rsbV1* gene encodes an anti–anti-sigma factor that is hypothesized to belong to a conserved partner switching mechanism in *Chlamydia* [33]. We first constructed the *rsbV1*::GII(*bla*) mutant (DFCT13) and verified the intron insertion using PCR (Fig. 3; Table 1) and sequencing of the *rsbV1*::GII(*bla*) locus. As predicted, the intron inserted into *rsbV1* in an anti-sense orientation between base pairs 28 and 29 (sequencing data results are shown in Figure S3). DFCT13 was then transformed with pDFTT3(*aadA*) to create an *rsbV1*::GII(*bla*), *incA*::GII(*aadA*) double-insertion mutant, DFCT16. PCR was used to map both intron-insertion sites (Fig. 4; Table 1) and the *rsbV1*::GII(*bla*) and *incA*::GII(*aadA*) loci were sequenced. The point of insertions in the double mutant matched the insertion sites for the respective single insertion mutants.

**Fig. 5:**
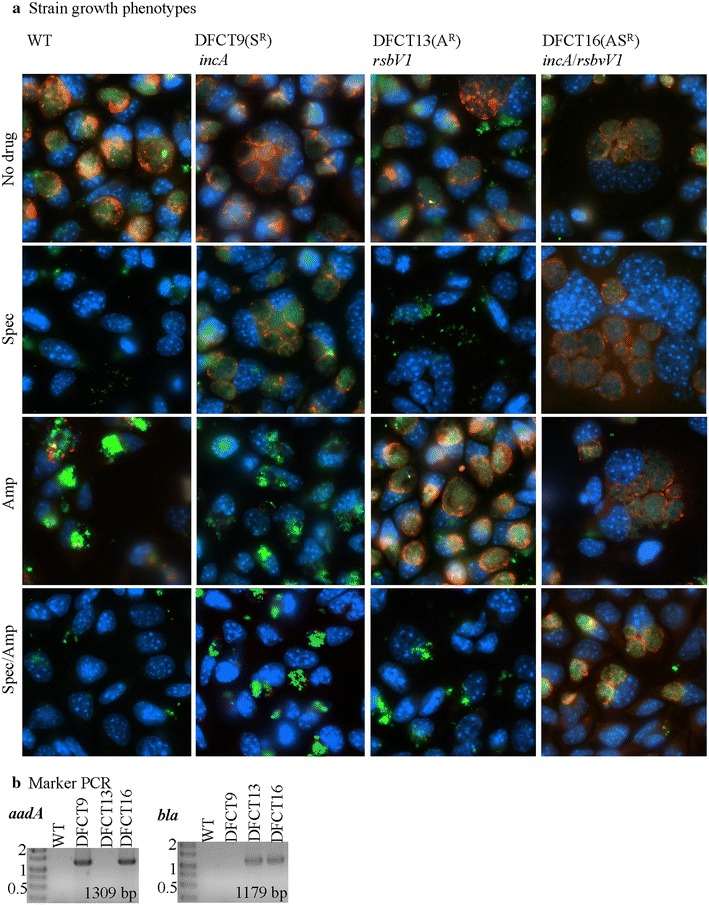
Growth phenotypes of DFCT9, DFCT13, and DFCT16 with and without ampicillin and/or spectinomycin. In panel **a**, L2 mouse cells were infected at an MOI ~5 with the wild type strain, DFCT9, DFCT13, or DFCT16 in the presence of no drug (*top row*), spectinomycin (*second row from top*), ampicillin (*third row from top*), or spectinomycin and ampicillin (*fourth row from top*). Resistance profiles and strain types are listed at the top of each row. Samples were processed for immunofluorescence microscopy at 24 h post infection. Cells were probed with anti-MOMP (*green*, marker for bacteria), anti-CT223 (*red*, inclusion membrane protein), and stained with DAPI (*blue*, DNA). Images were acquired at ×630 under oil-immersion and only the composite images are shown. In panel **b**, *bla* and *aadA* specific PCR was used to detect the resistance marker present in each strain. PCR products were visualized as described in Fig. 2

**Fig. 6:**
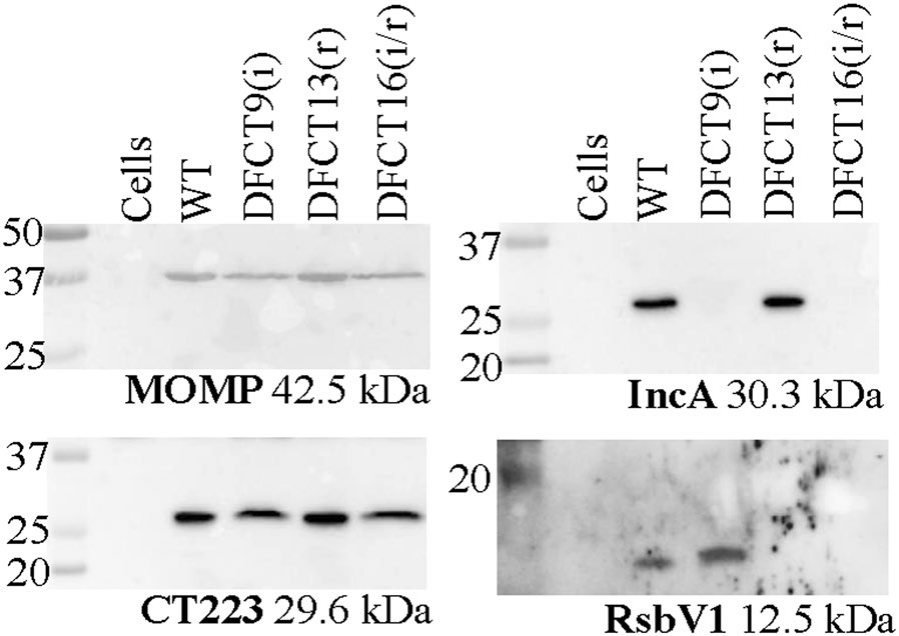
Western blot detection of IncA and RsbV1 in DFCT9, DFCT13, and DFCT16. Protein samples from cells infected in the absence of drug were run on SDS-PAGE, transferred to nitrocellulose, and probed with either anti-MOMP, anti-CT223, anti-IncA, or anti-RsbV1 antibodies. The anti-MOMP and anti-CT223 antibodies were used as controls to confirm the presence of bacteria and inclusion proteins, respectively, in each sample. Blots were then probed with HRP-conjugated secondary antibodies and visualized using a chemiluminescent substrate. Molecular weight markers (kDa) are shown to the left of each blot and the primary antibody used along with the predicted protein size are listed at the bottom of each blot. Contrast was adjusted (universally over the entire blot image) for the RsbV1 blot (to highlight band intensity). The (*i*) and (*r*) notations indicate the mutation(s) carried by the strain (*incA* and/or *rsbV1*)

Following molecular validation of the strains, all mutants were further assessed for phenotypes via inclusion morphology at 24 h post-infection using immunofluorescence microscopy and production of IncA and RsbV1 (Figs. 5; 6, respectively). All mutants grew in the presence of the appropriate antibiotics and were inhibited by antibiotics for which resistance markers were lacking. An anti-MOMP antibody was used as a marker for *C. trachomatis* and an anti-CT223 antibody was used as a marker for the inclusion (CT223 is an inclusion membrane protein [34]). In the presence of ampicillin, strains lacking the *bla* marker presented as enlarged, aberrant RBs within inclusions that were mostly negative for the inclusion membrane protein CT223 (as seen for the wild type strain and DFCT9 in Fig. 5a). Strains lacking the *aadA* marker formed small, punctate inclusions lacking CT223-staining (wild type strain and DFCT13) in the presence of spectinomycin (Fig. 5a). Aberrant RBs are observed under certain stress conditions, including beta-lactam treatment, and are thought to be viable, non-dividing bacteria [35–38]. Both IncA-null strains, DFCT9 and DFCT16, formed multiple inclusions consistent with the role of IncA in inclusion fusion. IncA-null status of the mutants was further confirmed using anti-IncA western blot analysis (Fig. 6). No obvious phenotype alterations were observed for the RsbV1-null strains compared to the wild type strain or the IncA-null strain (in the case of the double-mutant) under the conditions tested. Recent work by Thompson et al. demonstrates that an RsbV1-null strain shows minor growth defects and transcriptional differences that would not have been detected in our assay [39]. Western blot using an anti-RsbV1 antibody confirmed that the *rsbv1* mutants DFCT13 and DFCT16 do not produce RsbV1 (Fig. 6).

## Conclusions

Collectively, our results further expand the utility of the TargeTron system for mutagenesis in *Chlamydia* by allowing for: (1) complementation with existing shuttle vectors, (2) inactivation of multiple genes in the same bacterium and mutagenesis outside of the LGV serovar, and (3) use in vivo where long-term intron stability had not been previously assessed. The latter point is relevant to other pathogens in which the TargeTron system is employed as it alleviates the need for experimental conditions that could lead to disruption of the normal microflora due to antibiotic administration. In addition, we validated *aadA* as a resistance marker in *Chlamydia*. As spectinomycin is not a recommended drug for treatment of infections caused by *Chlamydia* spp., the *aadA* marker should be applicable as a selection marker across *Chlamydia*. Finally, the mutants created in this study will be useful for discerning the roles of *incA* and *rsbV1* in chlamydial physiology and virulence.

## Methods

*Bacterial and Cell CultureEscherichia coli* strain DH5α was used for all cloning procedures. *E. coli* were grown in either LB broth or on LB agar plates supplemented with antibiotics (100 μg/ml ampicillin, 100 μg/ml spectinomycin, and/or 20 μg/ml chloramphenicol) at 30 °C. Mouse L2 fibroblasts were used for cell culture experiments and passage of *C. trachomatis* L2 434/Bu. Cells were grown in DMEM supplemented with 10 % FBS at 37 °C with 5 % CO_2_. For routine growth of *C. trachomatis*, cells were grown until confluent and then infected with EBs via centrifugation at 545*g* for 1 hour. *Chlamydia*-infected cells were grown at 37 °C with 5 % CO_2_ in DMEM, 10 % FBS, 0.2 μg/ml cycloheximide, and 1× non-essential amino acids. EB stocks were titered using the inclusion forming unit assay (IFU) and stored in sucrose phosphate-buffered glutamic acid (SPG, 0.19 mM KH_2_PO_4_, 0.36 mM K_2_HPO_4_, 0.245 mM l-glutamic acid, 10.9 mM sucrose) at −80 °C. Strains created in this study and specific growth conditions are listed in supplementary Table 1.

*Construction of the intron donor vectors pDFTT3aadA and pDFTT6bla* The pDFTT3 vector (*incA*-targeted intron carrying the *bla* marker [20]) was used as the base vector for construction of pDFTT3*aadA* and pDFTT6*bla* (Figure S1). Phusion High-Fidelity PCR Master Mix (Thermo Scientific) was used for all PCR reactions and DNA was routinely analyzed using agarose gel electrophoresis and ethidium bromide staining. Primers were used at a concentration of 0.5 μM. DNA isolation (plasmids, PCR products, and gel-purified fragments) was performed using the respective GeneJET DNA kit (Thermo Scientific). All DNA constructs were sequenced by Macrogen USA.

To create pDFTT3*aadA*, the *aadA* gene from pAM238 [40] was PCR amplified with primers aadAF and aadAR (primer sequences are provided in supplementary Table 2). The PCR product was then digested with MluI and ligated into a similarly digested pDFTT3, effectively replacing the *bla* cassette with the *aadA* cassette (Figure S2). To create the *rsbV1*-targeted intron in pDFTT6*bla*, the *rsbV1* sequence was analyzed for insertion sites using the Sigma-Aldrich TargeTron algorithm. The predicted insertion site (TCCCTTGTAAATGAAGGATGCCTGTTTGGC—intron—CTTGTTCTTCTTTCT, antisense orientation, Figure S3) had an E-value of 0.75 and a score of 8.51. To re-target the intron, primers rsbV1 IBS, rsbV1 EBS1d, rsbV1 EBS2, and Univ were used in a PCR reaction with pDFTT3 as template to create the modified targeting region (PCR reaction conditions are specified in the TargeTron manual, Sigma-Aldrich). The PCR product was then digested with BsrGI and HindIII and ligated into a similarly digested pDFTT3. All ligation products were transformed into *E. coli* DH5α and transformants were selected on LB agar plates with the appropriate antibiotics.

*Construction of C. trachomatis mutants* Transformation of *C. trachomatis* and mutant selection with the GII(*bla*) vector was performed as described in Johnson and Fisher to create DFCT13 (*rsbV1*:GII[*bla*]) [20]. When using the GII(*aadA*) vector to create DFCT9 (*incA*::GII[*aadA*]) and DFCT16 (*incA*::GII[*aadA*], *rsbV1*::GII[*bla*]), spectinomycin was substituted for ampicillin and was used at 500 μg/ml for all selection steps. After multiple rounds of selection, transformants were titered and clones were obtained using a standard plaque assay. Plaque isolates were expanded in cell culture, tested for mycoplasma contamination using PCR, and stored in SPG. DFCT16 was created by transforming DFCT13 with pDFTT3*aadA* under spectinomycin selection.

All recombinant DNA experiments were performed in accordance with NIH section III-D-1-a and were approved by the Southern Illinois University recombinant DNA committee.

*Molecular analysis of DFCT9, DFCT13, and DFCT16* Genomic DNA was isolated using the DNeasy Blood & Tissue kit (Qiagen) and PCR was performed using Phusion High-Fidelity PCR Master Mix. PCR reactions were run with either 50 ng of genomic DNA or 1 ng of plasmid DNA as template. PCR products were separated on 1 % agarose gels, stained with ethidium bromide, and visualized using UV transillumination. The primer pairs used are listed in Table 1.

For DNA sequencing, the *incA* and *rsbV1* wild type and intron-disrupted loci were PCR amplified using primers incAF/incAR or V1F/V1R, respectively. PCR products were ligated into the pJET vector (Life Technologies) and transformants were grown for plasmid DNA isolation. Plasmid-inserts were sequenced by Macrogen USA using the primers pJET1_2F (CGACTCACTATAGGGAGAGCGGC) and pJET1_2R (AAGAACATCGATTTTCCATGGCAG). Sequence analysis was performed using Clone Manager (Scientific and Educational Software) and sequences were compared with the *C. trachomatis* L2 434/Bu genome (accession number: NC_010287).

*Phenotype analysis of DFCT9, DFCT13, and DFCT16* Confluent monolayers of L2 mouse cells grown in 24 well dishes (with or without glass coverslips) were mock-infected or infected using centrifugation with the wild type strain, DFCT9, DFCT13, or DFCT16 diluted in DMEM to an MOI of ~5. Following centrifugation, the medium was replaced with DMEM, 10 % FBS, 0.2 μg/ml cycloheximide, 1x non-essential amino acids, and antibiotics (5 μg/ml ampicillin and/or 500 μg/ml for spectinomycin). Infected cells were incubated at 37 °C, 5 % CO_2_.

Phase contrast light micrographs, immunofluorescent micrographs, and protein samples for western blot were obtained at 24 h post-infection. For immunofluorescence microscopy, cells were fixed and then probed with mouse monoclonal anti-CT223 antibodies (Dan Rockey, Oregon State University, 1:10) followed by goat anti-mouse IgG-Texas red conjugated antibodies (1:1000). Samples were then probed with FITC-conjugated mouse anti-MOMP antibodies (1:1000) and stained with DAPI to detect DNA. Fluorescent images were acquired using a Leica DM4000 fitted with a QImaging QiClick Mono camera and composite images were built using QImaging Software.

For western blot analysis, non-antibiotic treated samples were washed with phosphate buffered saline followed by addition of Laemmli buffer. Laemmli-treated samples were heated for 5 min at 95 °C and loaded into 12 % SDS-PAGE gels. Following electrophoresis, proteins were transferred to nitrocellulose for western blotting. Blots were blocked with 5 % milk Tris buffered saline (MTBS) and incubated overnight at 4 °C with either mouse anti-MOMP (Abcam, 1:1000), anti-IncA, anti-CT223 (both provided by Dan Rockey, Oregon State University, 1:250), or rabbit polyclonal anti-RsbV1 (this study, raised against the RsbV1 peptide KVFDSVNEALQALAKENS, performed by Thermo-Scientific Pierce, 1:200) antibodies diluted in MTBS. Blots were washed with 0.05 % tween Tris buffered saline (TTBS) and then incubated with HRP-conjugated anti-mouse IgG or anti-rabbit IgG antibodies diluted in MTBS. Prior to incubation with Immobilon Western Chemiluminescent HRP substrate (Millipore) the blots were washed with TTBS.

*Assessment of intron stability* in vivo Balb/C mice (8-weeks-old) were purchased from Harlan Laboratories (Indianapolis, IN). Seven days prior to infection with DFCT3 (*incA*::GII[*bla*]), mice were injected subcutaneously with 2.5 mg of progesterone (Depo-Provera, UpJohn), [41]. Five mice were then challenged intravaginally with 3x10^6^ IFU using a sterile pipette tip as previously described. No antibiotics were administered to the mice throughout the course of infection. Genital swabs were performed at 3 day intervals post infection and were used to measure EBs via the IFU assay. Swab samples also were used to verify intron presence using PCR and inclusion phenotype. To obtain enough material for these assays, swab samples were serially passaged in L2 mouse cells without ampicillin. All animal procedures were approved by the University of Arkansas Institutional Animal Care and Use Committee.

## Additional files

10.1186/s13104-015-1542-9 TargeTron vector maps and sequence files. Vector maps and Genbank sequence files were generated using Clone Manager from Scientific & Educational Software. The re-targeted DNA was cloned between the HindIII and BsrGI restriction sites. The N base pairs within the sequence files indicate the sequences re-written for gene specific targeting. The “mobile” region of the intron sequences are highlighted in red (GII[*aadA*]) or pink (GII[*bla*]).
10.1186/s13104-015-1542-9 Sequence map of the *incA*::GII(*aadA*) locus. The *incA*::GII(*aadA*) locus was PCR amplified from both DFCT9 and DFCT16 and cloned into pJET vectors for Sanger sequencing. The GII intron inserts into the *incA* open reading frame after position 108 (positon 1 is the A in ATG) resulting in a protein sequence differing from the wild type IncA at amino acid 37 and a stop codon at position 48. The intron sequence is highlighted in red. The entire intron sequence is not shown (the entire mobile intron sequence is shown in red in Figure S1B).
10.1186/s13104-015-1542-9 Sequence map of the *rsbV1*::GII(*bla*) locus. The *rsbV1*::GII(*bla*) locus was PCR amplified from both DFCT13 and DFCT16 and cloned into pJET for Sanger sequencing. The intron inserts into the *rsbV1* open reading frame after position 28 (positon 1 is the A in ATG) resulting in a protein sequence differing from the wild type RsbV1 at amino acid 11 and a stop codon at position 96. The the intron sequence is highlighted in pink. The entire intron sequence is not shown (the entire mobile intron sequence is shown in pink in Figure S1D).
10.1186/s13104-015-1542-9 Strains used in this study.
10.1186/s13104-015-1542-9 Primers used in this study.

## Abbreviations

GII: Group II intron
IFU: Inclusion forming unit
