# Supplementary material for: Use of aminoglycoside 3′ adenyltransferase as a selection marker for Chlamydia trachomatis intron-mutagenesis and in vivo intron stability
Source: BMC Res Notes. 2015 Oct 15;8:570. doi: 10.1186/s13104-015-1542-9 (PMC4606545; doi:10.1186/s13104-015-1542-9)
Supplement: Supplementary file 1 — 10.1186/s13104-015-1542-9 TargeTron vector maps and sequence files. Vector maps and Genbank sequence files were generated using Clone Manager from Scientific & Educational Software. The re-targeted DNA was cloned between the HindIII and BsrGI restriction sites. The N base pairs within the sequence files indicate the sequences re-written for gene specific targeting. The “mobile” region of the intron sequences are highlighted in red (GII[aadA]) or pink (GII[bla]). [file 13104_2015_1542_MOESM1_ESM.docx]

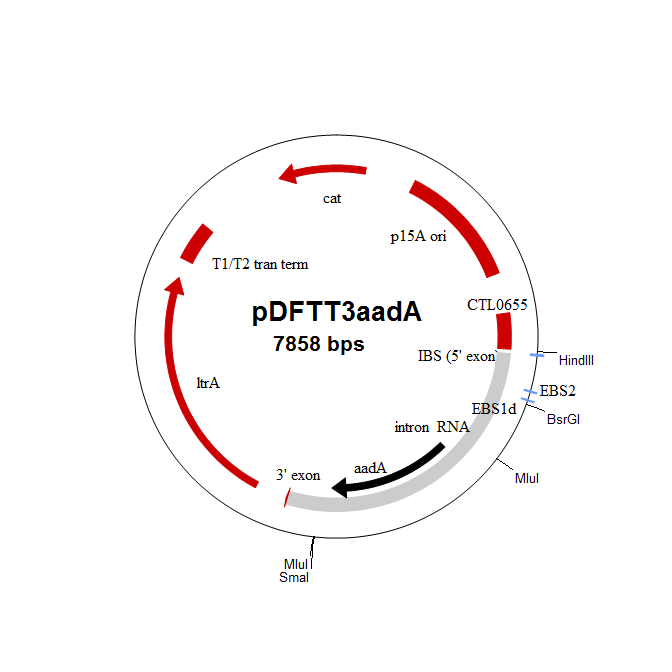
**Figure S1**

A) Map of pDFTT3*aadA*

B) Sequence file for pDFTT3*aadA*

LOCUS pDFTT3aadA 7858 bp

FEATURES Location/Qualifiers

CDS 585..1497

/gene="p15A ori"

CDS 1793..2055

/gene="CTL0655 promoter"

misc_feature 2075..2086

/gene="IBS (5' exon)"

/product="aaagaaCatagca"

CDS 2087..4298

/gene="intron RNA"

misc_signal 2087..4298

/label=insertion

/product="region inserted into target"

misc_feature 2306..2314

/gene="EBS2"

/product="gTTttcttc"

misc_feature 2363..2370

/gene="EBS1d"

/product="gctgctat"

CDS 2958..3968

/gene="aadA"

CDS 4298

/gene="d+1"

CDS 4299..4308

/gene="3' exon"

CDS 4545..6344

/gene="ltrA"

CDS 6480..6770

/gene="T1/T2 tran term"

CDS complement(join(7421..7858,1..219))

/gene="cat"

ORIGIN

1 gaattccgga tgagcattca tcaggcgggc aagaatgtga ataaaggccg gataaaactt

61 gtgcttattt ttctttacgg tctttaaaaa ggccgtaata tccagctgaa cggtctggtt

121 ataggtacat tgagcaactg actgaaatgc ctcaaaatgt tctttacgat gccattggga

181 tatatcaacg gtggtatatc cagtgatttt tttctccatt ttagcttcct tagctcctga

241 aaatctcgat aactcaaaaa atacgcccgg tagtgatctt atttcattat ggtgaaagtt

301 ggaacctctt acgtgccgat caacgtctca ttttcgccaa aagttggccc agggcttccc

361 ggtatcaaca gggacaccag gatttattta ttctgcgaag tgatcttccg tcacaggtat

421 ttattcggcg caaagtgcgt cgggtgatgc tgccaactta ctgatttagt gtatgatggt

481 gtttttgagg tgctccagtg gcttctgttt ctatcagctg tccctcctgt tcagctactg

541 acggggtggt gcgtaacggc aaaagcaccg ccggacatca gcgctagcgg agtgtatact

601 ggcttactat gttggcactg atgagggtgt cagtgaagtg cttcatgtgg caggagaaaa

661 aaggctgcac cggtgcgtca gcagaatatg tgatacagga tatattccgc ttcctcgctc

721 actgactcgc tacgctcggt cgttcgactg cggcgagcgg aaatggctta cgaacggggc

781 ggagatttcc tggaagatgc caggaagata cttaacaggg aagtgagagg gccgcggcaa

841 agccgttttt ccataggctc cgcccccctg acaagcatca cgaaatctga cgctcaaatc

901 agtggtggcg aaacccgaca ggactataaa gataccaggc gtttccccct ggcggctccc

961 tcgtgcgctc tcctgttcct gcctttcggt ttaccggtgt cattccgctg ttatggccgc

1021 gtttgtctca ttccacgcct gacactcagt tccgggtagg cagttcgctc caagctggac

1081 tgtatgcacg aaccccccgt tcagtccgac cgctgcgcct tatccggtaa ctatcgtctt

1141 gagtccaacc cggaaagaca tgcaaaagca ccactggcag cagccactgg taattgattt

1201 agaggagtta gtcttgaagt catgcgccgg ttaaggctaa actgaaagga caagttttgg

1261 tgactgcgct cctccaagcc agttacctcg gttcaaagag ttggtagctc agagaacctt

1321 cgaaaaaccg ccctgcaagg cggttttttc gttttcagag caagagatta cgcgcagacc

1381 aaaacgatct caagaagatc atcttattaa tcagataaaa tatttctagc tagatttcag

1441 tgcaatttat ctcttcaaat gtagcacctg aagtcagccc catacgatat aagttgtaat

1501 tctcatgttt gacagcttat catcgataag ctcaaggaga tggcgcccaa cagtcccccg

1561 gccacggggc ctgccaccat acccacgccg aaacaagcgc tcatgagccc gaagtggcga

1621 gcccgatctt ccccatcggt gatgtcggcg atataggcgc cagcaaccgc acctgtggcg

1681 ccggtgatgc cggccacgat gcgtccggcg tagaggatcg agatctcgat cccgcgaaat

1741 taatacgact cactataggg gaattgtgag cggataacaa ttcccctcta gaggtaccgg

1801 atcctccaaa ttattcctta catgaatttt ttgtctgagc gactttctcc cattgaaaaa

1861 gattttctta aacaaaacgt gctttacttc ttgcagaaaa atcggtagac ttgccgtttc

1921 gtctaggcag actcgtccgc gtcttttttc aaaactccct ttttaggaag tttttgaagg

1981 cgttcctcag attttcccga gttggaggag actggccggc actacaagct catatcaagg

2041 taaggaaaga tttccaagct tataattatc cttannnnnc nnnnnngtgc gcccagatag

2101 ggtgttaagt caagtagttt aaggtactac tctgtaagat aacacagaaa acagccaacc

2161 taaccgaaaa gcgaaagctg atacgggaac agagcacggt tggaaagcga tgagttacct

2221 aaagacaatc gggtacgact gagtcgcaat gttaatcaga tataaggtat aagttgtgtt

2281 tactgaacgc aagtttctaa tttcgnttnn nnnncgatag aggaaagtgt ctgaaacctc

2341 tagtacaaag aaaggtaagt tannnnnnnn gacttatctg ttatcaccac atttgtacaa

2401 tctgtaggag aacctatggg aacgaaacga aagcgatgcc gagaatctga atttaccaag

2461 acttaacact aactggggat accctaaaca agaatgccta atagaaagga ggaaaaaggc

2521 tatagcacta gagcttgaaa atcttgcaag ggtacggagt actcgtagta gtctgagaag

2581 ggtaacgccc tttacatggc aaaggggtac agttattgtg tactaaaatt aaaaattgat

2641 tagggaggaa aacctcaaaa tgaaaccaac aatggcaatt ttagaaagaa tcagtaaaaa

2701 ttcacaagaa aatatagacg aagtttttac aagactttat cgttatcttt tacgtccaga

2761 tatttattac gtggcgacgc gttgcctgac gatgcgtgga gaccgaaacc ttgcgctcgt

2821 tcgccagcca ggacagaaat gcctcgactt cgctgctgcc caaggttgcc gggtgacgca

2881 caccgtggaa acggatgaag gcacgaaccc agtggacata agcctgttcg gttcgtaagc

2941 tgtaatgcaa gtagcgtatg cgctcacgca actggtccag aaccttgacc gaacgcagcg

3001 gtggtaacgg cgcagtggcg gttttcatgg cttgttatga ctgttttttt ggggtacagt

3061 ctatgcctcg ggcatccaag cagcaagcgc gttacgccgt gggtcgatgt ttgatgttat

3121 ggagcagcaa cgatgttacg cagcagggca gtcgccctaa aacaaagtta aacatcatga

3181 gggaagcggt gatcgccgaa gtatcgactc aactatcaga ggtagttggc gtcatcgagc

3241 gccatctcga accgacgttg ctggccgtac atttgtacgg ctccgcagtg gatggcggcc

3301 tgaagccaca cagtgatatt gatttgctgg ttacggtgac cgtaaggctt gatgaaacaa

3361 cgcggcgagc tttgatcaac gaccttttgg aaacttcggc ttcccctgga gagagcgaga

3421 ttctccgcgc tgtagaagtc accattgttg tgcacgacga catcattccg tggcgttatc

3481 cagctaagcg cgaactgcaa tttggagaat ggcagcgcaa tgacattctt gcaggtatct

3541 tcgagccagc cacgatcgac attgatctgg ctatcttgct gacaaaagca agagaacata

3601 gcgttgcctt ggtaggtcca gcggcggagg aactctttga tccggttcct gaacaggatc

3661 tatttgaggc gctaaatgaa accttaacgc tatggaactc gccgcccgac tgggctggcg

3721 atgagcgaaa tgtagtgctt acgttgtccc gcatttggta cagcgcagta accggcaaaa

3781 tcgcgccgaa ggatgtcgct gccgactggg caatggagcg cctgccggcc cagtatcagc

3841 ccgtcatact tgaagctaga caggcttatc ttggacaaga agaagatcgc ttggcctcgc

3901 gcgcagatca gttggaagaa tttgtccact acgtgaaagg cgagatcacc aaggtagtcg

3961 gcaaataatg tctaacaatt cgttcaagcc gacgccgctt cgcggcgcgg cttaactcaa

4021 gcgttagatg cactaagcac ataattgctc acagccaaac tatcaggccc gggacgcgtt

4081 gggaaatggc aatgatagcg aaacaacgta aaactcttgt tgtatgcttt cattgtcatc

4141 gtcacgtgat tcataaacac aagtgaatgt cgacagtgaa tttttacgaa cgaacaataa

4201 cagagccgta tactccgaga ggggtacgta cggttcccga agagggtggt gcaaaccagt

4261 cacagtaatg tgaacaaggc ggtacctccc tacttcacca tatcattttc tgcagccccc

4321 tagaaataat tttgtttaac tttaagaagg agatatacat atatggctag atcgtccatt

4381 ccgacagcat cgccagtcac tatggcgtgc tgctagcgct atatgcgttg atgcaatttc

4441 tatgcactcg tagtagtctg agaagggtaa cgccctttac atggcaaagg ggtacagtta

4501 ttgtgtacta aaattaaaaa ttgattaggg aggaaaacct caaaatgaaa ccaacaatgg

4561 caattttaga aagaatcagt aaaaattcac aagaaaatat agacgaagtt tttacaagac

4621 tttatcgtta tcttttacgt ccagatattt attacgtggc gtatcaaaat ttatattcca

4681 ataaaggagc ttccacaaaa ggaatattag atgatacagc ggatggcttt agtgaagaaa

4741 aaataaaaaa gattattcaa tctttaaaag acggaactta ctatcctcaa cctgtacgaa

4801 gaatgtatat tgcaaaaaag aattctaaaa agatgagacc tttaggaatt ccaactttca

4861 cagataaatt gatccaagaa gctgtgagaa taattcttga atctatctat gaaccggtat

4921 tcgaagatgt gtctcacggt tttagacctc aacgaagctg tcacacagct ttgaaaacaa

4981 tcaaaagaga gtttggcggc gcaagatggt ttgtggaggg agatataaaa ggctgcttcg

5041 ataatataga ccacgttaca ctcattggac tcatcaatct taaaatcaaa gatatgaaaa

5101 tgagccaatt gatttataaa tttctaaaag caggttatct ggaaaactgg cagtatcaca

5161 aaacttacag cggaacacct caaggtggaa ttctatctcc tcttttggcc aacatctatc

5221 ttcatgaatt ggataagttt gttttacaac tcaaaatgaa gtttgaccga gaaagtccag

5281 aaagaataac acctgaatat cgggagctcc acaatgagat aaaaagaatt tctcaccgtc

5341 tcaagaagtt ggagggtgaa gaaaaagcta aagttctttt agaatatcaa gaaaaacgta

5401 aaagattacc cacactcccc tgtacctcac agacaaataa agtattgaaa tacgtccggt

5461 atgcggacga cttcattatc tctgttaaag gaagcaaaga ggactgtcaa tggataaaag

5521 aacaattaaa actttttatt cataacaagc taaaaatgga attgagtgaa gaaaaaacac

5581 tcatcacaca tagcagtcaa cccgctcgtt ttctgggata tgatatacga gtaaggagat

5641 ctggaacgat aaaacgatct ggtaaagtca aaaagagaac actcaatggg agtgtagaac

5701 tccttattcc tcttcaagac aaaattcgtc aatttatttt tgacaagaaa atagctatcc

5761 aaaagaaaga tagctcatgg tttccagttc acaggaaata tcttattcgt tcaacagact

5821 tagaaatcat cacaatttat aattctgaac tccgcgggat ttgtaattac tacggtctag

5881 caagtaattt taaccagctc aattattttg cttatcttat ggaatacagc tgtctaaaaa

5941 cgatagcctc caaacataag ggaacacttt caaaaaccat ttccatgttt aaagatggaa

6001 gtggttcgtg ggggatcccg tatgagataa agcaaggtaa gcagcgccgt tattttgcaa

6061 attttagtga atgtaaatcc ccttatcaat ttacggatga gataagtcaa gctcctgtat

6121 tgtatggcta tgcccggaat actcttgaaa acaggttaaa agctaaatgt tgtgaattat

6181 gtgggacgtc tgatgaaaat acttcctatg aaattcacca tgtcaataag gtcaaaaatc

6241 ttaaaggcaa agaaaaatgg gaaatggcaa tgatagcgaa acaacgtaaa actcttgttg

6301 tatgctttca ttgtcatcgt cacgtgattc ataaacacaa gtgaatgtcg agcacccgtt

6361 ctcggagcac tgtccgaccg ctttggccgc cgcccagtcc tgctcgcttc gctacttgga

6421 gccactatcg actacgcgat catggcgacc acacccgtcc tgtggatcgc caagctcgcc

6481 gatggtagtg tggggtctcc ccatgcgaga gtagggaact gccaggcatc aaataaaacg

6541 aaaggctcag tcgaaagact gggcctttcg ttttatctgt tgtttgtcgg tgaacgctct

6601 cctgagtagg acaaatccgc cgggagcgga tttgaacgtt gcgaagcaac ggcccggagg

6661 gtggcgggca ggacgcccgc cataaactgc caggcatcaa attaagcaga aggccatcct

6721 gacggatggc ctttttgcgt ttctacaaac tcttcctgtc gtcatatcta caagccatcc

6781 ccccacagat acggtaaact agcctcgttt ttgcatcagg aaagcagaac gccatgagcg

6841 gcctcatttc ttattctgag ttacaacagt ccgcaccgct gtccggtagc tccttccggt

6901 gggcgcgggg catgactatc gtcgccgcac ttatgactgt cttctttatc atgcaactcg

6961 taggacaggt gccggcagcg cccaacagtc ccccggccac ggggcctgcc accataccca

7021 cgccgaaaca agcgccctgc accattatgt tccggatctg catcgcagga tgctgctggc

7081 taccctgtgg aacacctaca tctgtattaa cgaagcgcta accgttttta tcaggctctg

7141 ggaggcagaa taaatgatca tatcgtcaat tattacctcc acggggagag cctgagcaaa

7201 ctggcctcag gcatttgaga agcacacggt cacactgctt ccggtagtca ataaaccggt

7261 aaaccagcaa tagacataag cggctattta acgaccctgc cctgaaccga cgaccgggtc

7321 gaatttgctt tcgaatttct gccattcatc cgcttattat cacttattca ggcgtagcac

7381 caggcgttta agggcaccaa taactgcctt aaaaaaatta cgccccgccc tgccactcat

7441 cgcagtactg ttgtaattca ttaagcattc tgccgacatg gaagccatca cagacggcat

7501 gatgaacctg aatcgccagc ggcatcagca ccttgtcgcc ttgcgtataa tatttgccca

7561 tggtgaaaac gggggcgaag aagttgtcca tattggccac gtttaaatca aaactggtga

7621 aactcaccca gggattggct gagacgaaaa acatattctc aataaaccct ttagggaaat

7681 aggccaggtt ttcaccgtaa cacgccacat cttgcgaata tatgtgtaga aactgccgga

7741 aatcgtcgtg gtattcactc cagagcgatg aaaacgtttc agtttgctca tggaaaacgg

7801 tgtaacaagg gtgaacacta tcccatatca ccagctcacc gtctttcatt gccatacg

//


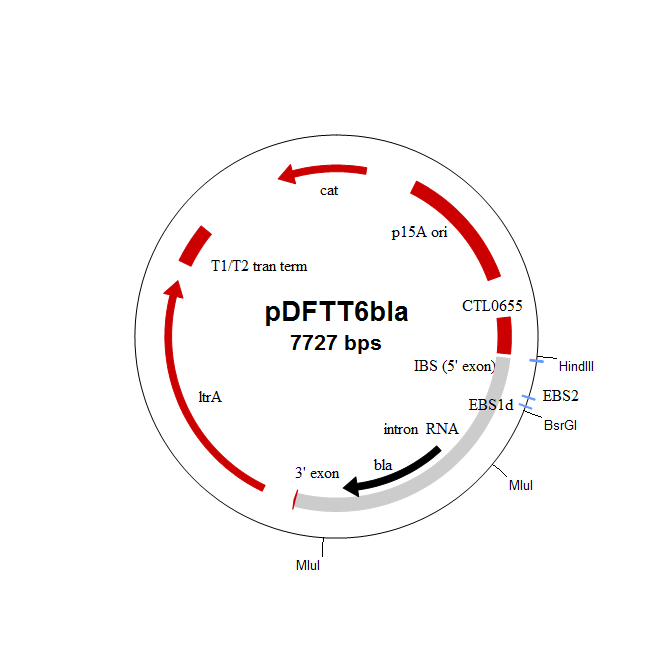
C) Map of pDFTT6bla

D) Sequence file for pDFTT3*bla*

LOCUS pDFTT6bla 7727 bp

FEATURES Location/Qualifiers

CDS 585..1497

/gene="p15A ori"

CDS 1793..2055

/gene="CTL0655 pro"

misc_feature 2075..2086

/gene="IBS (5' exon)"

/product="tgcctCtttggc"

CDS 2087..4167

/gene="intron RNA"

misc_signal 2087..4167

/label=insertion

/product="region inserted into target"

misc_feature 2306..2314

/gene="EBS2"

/product="gTTaggcat"

misc_feature 2363..2370

/gene="EBS1d"

/product="aggccaaa"

CDS 2950..3810

/gene="bla"

misc_signal 4167

/label=d+1

CDS 4168..4177

/gene="3' exon"

CDS 4414..6213

/gene="ltrA"

CDS 6349..6639

/gene="T1/T2 tran term"

CDS complement(join(7290..7727,1..219))

/gene="cat"

ORIGIN

1 gaattccgga tgagcattca tcaggcgggc aagaatgtga ataaaggccg gataaaactt

61 gtgcttattt ttctttacgg tctttaaaaa ggccgtaata tccagctgaa cggtctggtt

121 ataggtacat tgagcaactg actgaaatgc ctcaaaatgt tctttacgat gccattggga

181 tatatcaacg gtggtatatc cagtgatttt tttctccatt ttagcttcct tagctcctga

241 aaatctcgat aactcaaaaa atacgcccgg tagtgatctt atttcattat ggtgaaagtt

301 ggaacctctt acgtgccgat caacgtctca ttttcgccaa aagttggccc agggcttccc

361 ggtatcaaca gggacaccag gatttattta ttctgcgaag tgatcttccg tcacaggtat

421 ttattcggcg caaagtgcgt cgggtgatgc tgccaactta ctgatttagt gtatgatggt

481 gtttttgagg tgctccagtg gcttctgttt ctatcagctg tccctcctgt tcagctactg

541 acggggtggt gcgtaacggc aaaagcaccg ccggacatca gcgctagcgg agtgtatact

601 ggcttactat gttggcactg atgagggtgt cagtgaagtg cttcatgtgg caggagaaaa

661 aaggctgcac cggtgcgtca gcagaatatg tgatacagga tatattccgc ttcctcgctc

721 actgactcgc tacgctcggt cgttcgactg cggcgagcgg aaatggctta cgaacggggc

781 ggagatttcc tggaagatgc caggaagata cttaacaggg aagtgagagg gccgcggcaa

841 agccgttttt ccataggctc cgcccccctg acaagcatca cgaaatctga cgctcaaatc

901 agtggtggcg aaacccgaca ggactataaa gataccaggc gtttccccct ggcggctccc

961 tcgtgcgctc tcctgttcct gcctttcggt ttaccggtgt cattccgctg ttatggccgc

1021 gtttgtctca ttccacgcct gacactcagt tccgggtagg cagttcgctc caagctggac

1081 tgtatgcacg aaccccccgt tcagtccgac cgctgcgcct tatccggtaa ctatcgtctt

1141 gagtccaacc cggaaagaca tgcaaaagca ccactggcag cagccactgg taattgattt

1201 agaggagtta gtcttgaagt catgcgccgg ttaaggctaa actgaaagga caagttttgg

1261 tgactgcgct cctccaagcc agttacctcg gttcaaagag ttggtagctc agagaacctt

1321 cgaaaaaccg ccctgcaagg cggttttttc gttttcagag caagagatta cgcgcagacc

1381 aaaacgatct caagaagatc atcttattaa tcagataaaa tatttctagc tagatttcag

1441 tgcaatttat ctcttcaaat gtagcacctg aagtcagccc catacgatat aagttgtaat

1501 tctcatgttt gacagcttat catcgataag ctcaaggaga tggcgcccaa cagtcccccg

1561 gccacggggc ctgccaccat acccacgccg aaacaagcgc tcatgagccc gaagtggcga

1621 gcccgatctt ccccatcggt gatgtcggcg atataggcgc cagcaaccgc acctgtggcg

1681 ccggtgatgc cggccacgat gcgtccggcg tagaggatcg agatctcgat cccgcgaaat

1741 taatacgact cactataggg gaattgtgag cggataacaa ttcccctcta gaggtaccgg

1801 atcctccaaa ttattcctta catgaatttt ttgtctgagc gactttctcc cattgaaaaa

1861 gattttctta aacaaaacgt gctttacttc ttgcagaaaa atcggtagac ttgccgtttc

1921 gtctaggcag actcgtccgc gtcttttttc aaaactccct ttttaggaag tttttgaagg

1981 cgttcctcag attttcccga gttggaggag actggccggc actacaagct catatcaagg

2041 taaggaaaga tttccaagct tataattatc cttannnnnc nnnnnngtgc gcccagatag

2101 ggtgttaagt caagtagttt aaggtactac tctgtaagat aacacagaaa acagccaacc

2161 taaccgaaaa gcgaaagctg atacgggaac agagcacggt tggaaagcga tgagttacct

2221 aaagacaatc gggtacgact gagtcgcaat gttaatcaga tataaggtat aagttgtgtt

2281 tactgaacgc aagtttctaa tttcgnttnn nnnncgatag aggaaagtgt ctgaaacctc

2341 tagtacaaag aaaggtaagt tannnnnnnn gacttatctg ttatcaccac atttgtacaa

2401 tctgtaggag aacctatggg aacgaaacga aagcgatgcc gagaatctga atttaccaag

2461 acttaacact aactggggat accctaaaca agaatgccta atagaaagga ggaaaaaggc

2521 tatagcacta gagcttgaaa atcttgcaag ggtacggagt actcgtagta gtctgagaag

2581 ggtaacgccc tttacatggc aaaggggtac agttattgtg tactaaaatt aaaaattgat

2641 tagggaggaa aacctcaaaa tgaaaccaac aatggcaatt ttagaaagaa tcagtaaaaa

2701 ttcacaagaa aatatagacg aagtttttac aagactttat cgttatcttt tacgtccaga

2761 tatttattac gtggcgacgc gtaggttaat gtcatgataa taatggtttc ttagacgtca

2821 ggtggcactt ttcggggaaa tgtgcgcgga acccctattt gtttattttt ctaaatacat

2881 tcaaatatgt atccgctcat gagacaataa ccctgataaa tgcttcaata atattgaaaa

2941 aggaagagta tgagtattca acatttccgt gtcgccctta ttcccttttt tgcggcattt

3001 tgccttcctg tttttgctca cccagaaacg ctggtgaaag taaaagatgc tgaagatcag

3061 ttgggtgcac gagtgggtta catcgaactg gatctcaaca gcggtaagat ccttgagagt

3121 tttcgccccg aagaacgttt tccaatgatg agcactttta aagttctgct atgtggcgcg

3181 gtattatccc gtattgacgc cgggcaagag caactcggtc gccgcataca ctattctcag

3241 aatgacttgg ttgagtactc accagtcaca gaaaagcatc ttacggatgg catgacagta

3301 agagaattat gcagtgctgc cataaccatg agtgataaca ctgcggccaa cttacttctg

3361 acaacgatcg gaggaccgaa ggagctaacc gcttttttgc acaacatggg ggatcatgta

3421 actcgccttg atcgttggga accggagctg aatgaagcca taccaaacga cgagcgtgac

3481 accacgatgc ctgtagcaat ggcaacaacg ttgcgcaaac tattaactgg cgaactactt

3541 actctagctt cccggcaaca attaatagac tggatggagg cggataaagt tgcaggacca

3601 cttctgcgct cggcccttcc ggctggctgg tttattgctg ataaatctgg agccggtgag

3661 cgtgggtctc gcggtatcat tgcagcactg gggccagatg gtaagccctc ccgtatcgta

3721 gttatctaca cgacggggag tcaggcaact atggatgaac gaaatagaca gatcgctgag

3781 ataggtgcct cactgattaa gcattggtaa ctgtcagacc aagtttactc atatatactt

3841 tagattgatt taaaacttca tttttaattt aaaaggatct aggtgaagat cctttttgat

3901 aatctcatga ccaaaatccc ttaacgtgag ttttcgttcc acacgcgttg ggaaatggca

3961 atgatagcga aacaacgtaa aactcttgtt gtatgctttc attgtcatcg tcacgtgatt

4021 cataaacaca agtgaatgtc gacagtgaat ttttacgaac gaacaataac agagccgtat

4081 actccgagag gggtacgtac ggttcccgaa gagggtggtg caaaccagtc acagtaatgt

4141 gaacaaggcg gtacctccct acttcaccat atcattttct gcagccccct agaaataatt

4201 ttgtttaact ttaagaagga gatatacata tatggctaga tcgtccattc cgacagcatc

4261 gccagtcact atggcgtgct gctagcgcta tatgcgttga tgcaatttct atgcactcgt

4321 agtagtctga gaagggtaac gccctttaca tggcaaaggg gtacagttat tgtgtactaa

4381 aattaaaaat tgattaggga ggaaaacctc aaaatgaaac caacaatggc aattttagaa

4441 agaatcagta aaaattcaca agaaaatata gacgaagttt ttacaagact ttatcgttat

4501 cttttacgtc cagatattta ttacgtggcg tatcaaaatt tatattccaa taaaggagct

4561 tccacaaaag gaatattaga tgatacagcg gatggcttta gtgaagaaaa aataaaaaag

4621 attattcaat ctttaaaaga cggaacttac tatcctcaac ctgtacgaag aatgtatatt

4681 gcaaaaaaga attctaaaaa gatgagacct ttaggaattc caactttcac agataaattg

4741 atccaagaag ctgtgagaat aattcttgaa tctatctatg aaccggtatt cgaagatgtg

4801 tctcacggtt ttagacctca acgaagctgt cacacagctt tgaaaacaat caaaagagag

4861 tttggcggcg caagatggtt tgtggaggga gatataaaag gctgcttcga taatatagac

4921 cacgttacac tcattggact catcaatctt aaaatcaaag atatgaaaat gagccaattg

4981 atttataaat ttctaaaagc aggttatctg gaaaactggc agtatcacaa aacttacagc

5041 ggaacacctc aaggtggaat tctatctcct cttttggcca acatctatct tcatgaattg

5101 gataagtttg ttttacaact caaaatgaag tttgaccgag aaagtccaga aagaataaca

5161 cctgaatatc gggagctcca caatgagata aaaagaattt ctcaccgtct caagaagttg

5221 gagggtgaag aaaaagctaa agttctttta gaatatcaag aaaaacgtaa aagattaccc

5281 acactcccct gtacctcaca gacaaataaa gtattgaaat acgtccggta tgcggacgac

5341 ttcattatct ctgttaaagg aagcaaagag gactgtcaat ggataaaaga acaattaaaa

5401 ctttttattc ataacaagct aaaaatggaa ttgagtgaag aaaaaacact catcacacat

5461 agcagtcaac ccgctcgttt tctgggatat gatatacgag taaggagatc tggaacgata

5521 aaacgatctg gtaaagtcaa aaagagaaca ctcaatggga gtgtagaact ccttattcct

5581 cttcaagaca aaattcgtca atttattttt gacaagaaaa tagctatcca aaagaaagat

5641 agctcatggt ttccagttca caggaaatat cttattcgtt caacagactt agaaatcatc

5701 acaatttata attctgaact ccgcgggatt tgtaattact acggtctagc aagtaatttt

5761 aaccagctca attattttgc ttatcttatg gaatacagct gtctaaaaac gatagcctcc

5821 aaacataagg gaacactttc aaaaaccatt tccatgttta aagatggaag tggttcgtgg

5881 gggatcccgt atgagataaa gcaaggtaag cagcgccgtt attttgcaaa ttttagtgaa

5941 tgtaaatccc cttatcaatt tacggatgag ataagtcaag ctcctgtatt gtatggctat

6001 gcccggaata ctcttgaaaa caggttaaaa gctaaatgtt gtgaattatg tgggacgtct

6061 gatgaaaata cttcctatga aattcaccat gtcaataagg tcaaaaatct taaaggcaaa

6121 gaaaaatggg aaatggcaat gatagcgaaa caacgtaaaa ctcttgttgt atgctttcat

6181 tgtcatcgtc acgtgattca taaacacaag tgaatgtcga gcacccgttc tcggagcact

6241 gtccgaccgc tttggccgcc gcccagtcct gctcgcttcg ctacttggag ccactatcga

6301 ctacgcgatc atggcgacca cacccgtcct gtggatcgcc aagctcgccg atggtagtgt

6361 ggggtctccc catgcgagag tagggaactg ccaggcatca aataaaacga aaggctcagt

6421 cgaaagactg ggcctttcgt tttatctgtt gtttgtcggt gaacgctctc ctgagtagga

6481 caaatccgcc gggagcggat ttgaacgttg cgaagcaacg gcccggaggg tggcgggcag

6541 gacgcccgcc ataaactgcc aggcatcaaa ttaagcagaa ggccatcctg acggatggcc

6601 tttttgcgtt tctacaaact cttcctgtcg tcatatctac aagccatccc cccacagata

6661 cggtaaacta gcctcgtttt tgcatcagga aagcagaacg ccatgagcgg cctcatttct

6721 tattctgagt tacaacagtc cgcaccgctg tccggtagct ccttccggtg ggcgcggggc

6781 atgactatcg tcgccgcact tatgactgtc ttctttatca tgcaactcgt aggacaggtg

6841 ccggcagcgc ccaacagtcc cccggccacg gggcctgcca ccatacccac gccgaaacaa

6901 gcgccctgca ccattatgtt ccggatctgc atcgcaggat gctgctggct accctgtgga

6961 acacctacat ctgtattaac gaagcgctaa ccgtttttat caggctctgg gaggcagaat

7021 aaatgatcat atcgtcaatt attacctcca cggggagagc ctgagcaaac tggcctcagg

7081 catttgagaa gcacacggtc acactgcttc cggtagtcaa taaaccggta aaccagcaat

7141 agacataagc ggctatttaa cgaccctgcc ctgaaccgac gaccgggtcg aatttgcttt

7201 cgaatttctg ccattcatcc gcttattatc acttattcag gcgtagcacc aggcgtttaa

7261 gggcaccaat aactgcctta aaaaaattac gccccgccct gccactcatc gcagtactgt

7321 tgtaattcat taagcattct gccgacatgg aagccatcac agacggcatg atgaacctga

7381 atcgccagcg gcatcagcac cttgtcgcct tgcgtataat atttgcccat ggtgaaaacg

7441 ggggcgaaga agttgtccat attggccacg tttaaatcaa aactggtgaa actcacccag

7501 ggattggctg agacgaaaaa catattctca ataaaccctt tagggaaata ggccaggttt

7561 tcaccgtaac acgccacatc ttgcgaatat atgtgtagaa actgccggaa atcgtcgtgg

7621 tattcactcc agagcgatga aaacgtttca gtttgctcat ggaaaacggt gtaacaaggg

7681 tgaacactat cccatatcac cagctcaccg tctttcattg ccatacg

//

**Figure S1. TargeTron vector maps and sequence files.** Vector maps and Genbank sequence files were generated using Clone Manager from Scientific & Educational Software. The re-targeted DNA was cloned between the HindIII and BsrGI restriction sites. The N base pairs within the sequence files indicate the sequences re-written for gene specific targeting. The “mobile” region of the intron sequences are highlighted in red (GII[*aadA*]) or pink (GII[*bla*]).
