# Supplementary material for: Use of aminoglycoside 3′ adenyltransferase as a selection marker for Chlamydia trachomatis intron-mutagenesis and in vivo intron stability
Source: BMC Res Notes. 2015 Oct 15;8:570. doi: 10.1186/s13104-015-1542-9 (PMC4606545; doi:10.1186/s13104-015-1542-9)
Supplement: Supplementary file 2 — 10.1186/s13104-015-1542-9 Sequence map of the incA::GII(aadA) locus. The incA::GII(aadA) locus was PCR amplified from both DFCT9 and DFCT16 and cloned into pJET vectors for Sanger sequencing. The GII intron inserts into the incA open reading frame after position 108 (positon 1 is the A in ATG) resulting in a protein sequence differing from the wild type IncA at amino acid 37 and a stop codon at position 48. The intron sequence is highlighted in red. The entire intron sequence is not shown (the entire mobile intron sequence is shown in red in Figure S1B). [file 13104_2015_1542_MOESM2_ESM.docx]

**Figure S2**

**
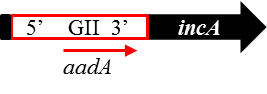
**

10 20 30 40 50 60 70 80

....|....|....|....|....|....|....|....|....|....|....|....|....|....|....|....|

**ATGACAACGCCTACTCTAATCGTGACTCCTCCATCTCCCCCTGCACCTTCCTACTCAGCCAATCGCGTACCTCAACCTTC**

M T T P T L I V T P P S P P A P S Y S A N R V P Q P S

90 100 110 120 130 140 150 160

....|....|....|....|....|....|....|....|....|....|....|....|....|....|....|....|

**TTTGATGGACAAAATTAAGAAAATAGCAGTGCGCCCAGATAGGGTGTTAAGTCAAGTAGTTTAAGGTACTACTCTGTAAG**

L M D K I K K I A V R P D R V L S Q V V *

**Intron Sequence...**

2250 2260 2270 2280 2290 2300 2310 2320

....|....|....|....|....|....|....|....|....|....|....|....|....|....|....|....|

**GAGGGGTACGTACGGTTCCCGAAGAGGGTGGTGCAAACCAGTCACAGTAATGTGAACAAGGCGGTACCTCCCTACTTCAC**

2330 2340 2350 2360 2370 2380 2390 2400

....|....|....|....|....|....|....|....|....|....|....|....|....|....|....|....|

**GCCATTGCCTCCCTAATTCTTATAGGCACAATAGGCTTTTTAGCTCTTTTGGGACATCTTGTTGGCTTTCTGATCGCTCC**

2410 2420 2430 2440 2450 2460 2470 2480

....|....|....|....|....|....|....|....|....|....|....|....|....|....|....|....|

**ACAAATCACTATTGTTCTTCTTGCCCTATTCATTATCTCATTAGCAGGGAATGCTCTTTATCTACAGAAAACCGCTAATC**

2490 2500 2510 2520 2530 2540 2550 2560

....|....|....|....|....|....|....|....|....|....|....|....|....|....|....|....|

**TACATCTATACCAGGATCTGCAAAGAGAAGTTGGGTCTCTAAAAGAAATTAATTTCATGCTGAGCGTTCTACAGAAAGAA**

2570 2580 2590 2600 2610 2620 2630 2640

....|....|....|....|....|....|....|....|....|....|....|....|....|....|....|....|

**TTTCTTCATTTATCTAAAGAATTTGCAACGACATCTAAAGACCTCTCTGCTGTATCTCAAGATTTTTATTCTTGTTTGCA**

2650 2660 2670 2680 2690 2700 2710 2720

....|....|....|....|....|....|....|....|....|....|....|....|....|....|....|....|

**AGGATTTAGAGATAACTATAAAGGTTTTGAATCTCTTTTGGATGAGTATAAAAACTCTACAGAAGAAATGCGCAAACTTT**

2730 2740 2750 2760 2770 2780 2790 2800

....|....|....|....|....|....|....|....|....|....|....|....|....|....|....|....|

**TTTCGCAAGAAATCATAGCAGATCTTAAAGGCTCTGTTGCCTCATTAAGAGAGGAAATCCGATTCCTAACCCCATTAGCA**

2810 2820 2830 2840 2850 2860 2870 2880

....|....|....|....|....|....|....|....|....|....|....|....|....|....|....|....|

**GAAGAAGTTCGCCGATTAGCGCATAACCAGCAATCATTAACAGTGGTTATTGAAGAATTAAAAACAATTCGTGATAGCTT**

2890 2900 2910 2920 2930 2940 2950 2960

....|....|....|....|....|....|....|....|....|....|....|....|....|....|....|....|

**ACGAGATGAAATTGGACAACTTTCACAACTTTCTAAAACTCTTACCAGTCAAATTGCATTACAACGAAAAGAGAGCTCAG**

2970 2980 2990 3000 3010 3020 3030

....|....|....|....|....|....|....|....|....|....|....|....|....|....|....

**ATCTGTGTTCCCAGATAAGAGAGACGCTCTCCTCCCCCAGAAAGTCTGCATCACCCTCTACAAAAAGCTCCTAG**

**Figure S2. Sequence map of the *incA*::GII(*aadA*) locus.** The *incA*::GII(*aadA*) locus was PCR amplified from both DFCT9 and DFCT16 and cloned into pJET vectors for Sanger sequencing. The GII intron inserts into the *incA* open reading frame after position 108 (positon 1 is the A in ATG) resulting in a protein sequence differing from the wild type IncA at amino acid 37 and a stop codon at position 48. The intron sequence is highlighted in red. The entire intron sequence is not shown (the entire mobile intron sequence is shown in red in Figure S1B).
