# Supplementary material for: Use of aminoglycoside 3′ adenyltransferase as a selection marker for Chlamydia trachomatis intron-mutagenesis and in vivo intron stability
Source: BMC Res Notes. 2015 Oct 15;8:570. doi: 10.1186/s13104-015-1542-9 (PMC4606545; doi:10.1186/s13104-015-1542-9)
Supplement: Supplementary file 3 — 10.1186/s13104-015-1542-9 Sequence map of the rsbV1::GII(bla) locus. The rsbV1::GII(bla) locus was PCR amplified from both DFCT13 and DFCT16 and cloned into pJET for Sanger sequencing. The intron inserts into the rsbV1 open reading frame after position 28 (positon 1 is the A in ATG) resulting in a protein sequence differing from the wild type RsbV1 at amino acid 11 and a stop codon at position 96. The the intron sequence is highlighted in pink. The entire intron sequence is not shown (the entire mobile intron sequence is shown in pink in Figure S1D). [file 13104_2015_1542_MOESM3_ESM.docx]

**Figure S3**


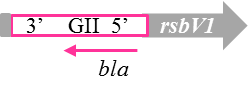


10 20 30 40 50 60 70 80

....|....|....|....|....|....|....|....|....|....|....|....|....|....|....|....|

**ATGAGTAACTTTCAGAAAGAAGAACAAGGTGAAGTAGGGAGGTACCGCCTTGTTCACATTACTGTGACTGGTTTGCACCA**

M S N F Q K E E Q G E V G R Y R L V H I T V T G L H H

90 100 110 120 130 140 150 160

....|....|....|....|....|....|....|....|....|....|....|....|....|....|....|....|

**CCCTCTTCGGGAACCGTACGTACCCCTCTCGGAGTATACGGCTCTGTTATTGTTCGTTCGTAAAAATTCACTGTCGACAT**

P L R E P Y V P L S E Y T A L L L F V R K N S L S T

170 180 190 200 210 220 230 240

....|....|....|....|....|....|....|....|....|....|....|....|....|....|....|....|

**TCACTTGTGTTTATGAATCACGTGACGATGACAATGAAAGCATACAACAAGAGTTTTACGTTGTTTCGCTATCATTGCCA**

F T C V Y E S R D D D N E S I Q Q E F Y V V S L S L P

250 260 270 280 290 300 310 320

....|....|....|....|....|....|....|....|....|....|....|....|....|....|....|....|

**TTTCCCAACGCGTGTGGAACGAAAACTCACGTTAAGGGATTTTGGTCATGAGATTATCAAAAAGGATCTTCACCTAGATC**

F P N A C G T K T H V K G F W S *

**Intron Sequence...**

2090 2100 2110 2120 2130 2140 2150 2160

....|....|....|....|....|....|....|....|....|....|....|....|....|....|....|....|

**ACTTGACTTAACACCCTATCTGGGCGCACGCCAAACAGGCATCCTTCATTTACAAGGGAAACTCGATGGTGTCTCCTCTC**

2170 2180 2190 2200 2210 2220 2230 2240

....|....|....|....|....|....|....|....|....|....|....|....|....|....|....|....|

**CAGCTGTACAAGAAAGTATTTCTGAATCTCTTTCCAATGGCATGAAAAACATTATTCTGGACTGCGGAGATTTAGATTAC**

2250 2260 2270 2280 2290 2300 2310 2320

....|....|....|....|....|....|....|....|....|....|....|....|....|....|....|....|

**ATATCTAGTGCAGGTATCCGCGTGCTTTTACAAAGCTATCACCAAGTAGGGAAAAATGCGGGGAAAATTGCTCTCACTTC**

2330 2340 2350 2360 2370 2380 2390 2400

....|....|....|....|....|....|....|....|....|....|....|....|....|....|....|....|

**TGTCTCTAAAACAGTGGAACAGACTCTTTATGTCACAGGCTTTTTGTCTTATTTTAAGGTCTTCGACTCTGTGAATGAAG**

2410 2420 2430

....|....|....|....|....|....|..

**CTCTACAAGCTTTAGCAAAAGAAAACAGTTAA**

**Figure S3. Sequence map of the *rsbV1*::GII(*bla*) locus.** The *rsbV1*::GII(*bla*) locus was PCR amplified from both DFCT13 and DFCT16 and cloned into pJET for Sanger sequencing. The intron inserts into the *rsbV1* open reading frame after position 28 (positon 1 is the A in ATG) resulting in a protein sequence differing from the wild type RsbV1 at amino acid 11 and a stop codon at position 96. The the intron sequence is highlighted in pink. The entire intron sequence is not shown (the entire mobile intron sequence is shown in pink in Figure S1D).
