# Supplementary material for: Use of aminoglycoside 3′ adenyltransferase as a selection marker for Chlamydia trachomatis intron-mutagenesis and in vivo intron stability
Source: BMC Res Notes. 2015 Oct 15;8:570. doi: 10.1186/s13104-015-1542-9 (PMC4606545; doi:10.1186/s13104-015-1542-9)
Supplement: Supplementary file 4 — 10.1186/s13104-015-1542-9 Strains used in this study. [file 13104_2015_1542_MOESM4_ESM.docx]

**Table S1. Strains used in this study.**

| Strain | Relevant Genotype | Notes | Reference |
| --- | --- | --- | --- |
| L2 |  | Mouse fibroblast |  |
| *E. coli* DH5α |  | Used for routine cloning |  |
| DF583 | pDFTT3*aadA* | *E. coli* DH5α, 100 μg/ml spectinomycin, 20 μg/ml chloramphenicol | this study |
| DF567 | pDFTT6*bla* | *E. coli* DH5α, 100 μg/ml ampicillin 20 μg/ml chloramphenicol | this study |
| *C. trachomatis* L2 434/Bu |  |  |  |
| DFCT3 | *incA*::GII(*bla*) | *C. trachomatis* L2 434/Bu, 5 μg/ml ampicillin | [[20]](#_ENREF_16) |
| DFCT9 | *incA*::GII(*aadA*) | *C. trachomatis* L2 434/Bu, 500 μg/ml spectinomycin | this study |
| DFCT13 | *rsbV1*::GII(*bla*) | *C. trachomatis* L2 434/Bu, 5 μg/ml ampicillin | this study |
| DFCT16 | *incA*::GII(*aadA*), *rsbV1*::GII(*bla*) | *C. trachomatis* L2 434/Bu, 5 μg/ml ampicillin and 500 μg/ml spectinomycin | this study |
