# Supplementary material for: Use of aminoglycoside 3′ adenyltransferase as a selection marker for Chlamydia trachomatis intron-mutagenesis and in vivo intron stability
Source: BMC Res Notes. 2015 Oct 15;8:570. doi: 10.1186/s13104-015-1542-9 (PMC4606545; doi:10.1186/s13104-015-1542-9)
Supplement: Supplementary file 5 — 10.1186/s13104-015-1542-9 Primers used in this study. [file 13104_2015_1542_MOESM5_ESM.docx]

**Table S2. Primers used in this study.**

| Primer | Sequence 5' to 3' | Notes |
| --- | --- | --- |
| V1F | CCTATCCCAATGAGTAACTTTC |  |
| V1R | GCAGGTCGACTTAACTGTTTTCTTTTGCTAAAG |  |
| V1R2 | AAGCACGCGGATACCTGCAC |  |
| incAF | ACCTTCCTACTCAGCCAATC |  |
| incAR | AATCGGCGAACTTCTTCTGC |  |
| hyp08F | CTCGTAATATGCAAGAGCATTGTAAG |  |
| hyp08R | GGCCGCAGAAGATATTCTGAAG |  |
| GIIF | AGCGATGCCGAGAATCTG |  |
| GIIR | TCTCGGAGTATACGGCTCTG |  |
| catF | GAAGTGAGGCATATGGAGAAAAAAATCACTGGATATAC |  |
| catR | ATGTCATCCCTCGAGTTACGCCCCGCCCTG |  |
| blaF1 | TCGCACGCGTAGGTTAATGTCATGATAATAATGG |  |
| blaR1 | GACACGCGTGTGGAACGAAAACTCACG |  |
| blaR2 | ACCTATCTCAGCGATCTGTC |  |
| aadAR | TCTACGCGTTGCCTGACGATGCGTGGAG | MluI site (underlined) |
| aadAF | GTAACGCGT*CCCGGG*CCTGATAGTTTGGCTGTGAG | MluI site (underlined) and SmaI site (underlined and in italics) |
| rsbV1 IBS | AAAAAAGCTTATAATTATCCTTA***TGCCT***C***TTTGGC***GTGCGCCCAGATAGGGTG | targeting primers, retargeting sequences in bold/italics, BsrgI site underlined |
| rbsV1 EBS1d | CAGATTGTACAAATGTGGTGATAACAGATAAGTC***TTTGGCCT***TAACTTACCTTTCTTTGT | targeting primers, retargeting sequences in bold/italics, HindIII site underlined |
| rsbV1 EBS2 | TGAACGCAAGTTTCTAATTTCG***G***TT***AGGCAT***CGATAGAGGAAAGTGTCT | targeting primers, , retargeting sequences in bold/italics |
| Univ | CGAAATTAGAAACTTGCGTTCAGTAAAC | targeting primers |
